# Low‐Dose vs. Standard Care Iv Human Albumin During Large‐Volume Paracentesis in Patients With Liver Cirrhosis: A Systematic Review

**DOI:** 10.1111/liv.70621

**Published:** 2026-03-26

**Authors:** Kimberly K. I. M. Bot, Roebi Heus, Joost P. H. Drenth, Marten A. Lantinga

**Affiliations:** ^1^ Department of Gastroenterology and Hepatology, Amsterdam UMC University of Amsterdam, Amsterdam Gastroenterology Endocrinology Metabolism Amsterdam the Netherlands; ^2^ European Reference Network on Hepatological Diseases (ERN RARE‐LIVER) Hamburg Germany

**Keywords:** ascites, human albumin, portal hypertension

## Abstract

Cirrhosis is a global health concern, with ascites being the most prevalent decompensating event. Intravenous (IV) human albumin (HA) is administered during large‐volume paracentesis (LVP) to prevent complications. The standard (6–8 g HA/L ascites) is empirically chosen, potentially leading to overtreatment and added costs. We reviewed evidence comparing the efficacy and safety of reduced versus standard‐care HA dosing during LVP. We searched PubMed, EMBASE, Cochrane Library, CINAHL, and Scopus (up to January 31, 2025). We included RCTs and cohort studies in adults with cirrhosis undergoing LVP, comparing low‐dose (≤ 6 g/L or ≤ 20 g total) to standard‐dose (> 6 g/L or > 20 g total) HA. Outcomes included paracentesis‐induced circulatory dysfunction (PICD), renal impairment, hyponatremia, other decompensating events, and survival. Quality assessment used Cochrane risk‐of‐bias tools and Newcastle–Ottawa Scale. Five studies (three full‐text, two abstracts) met inclusion criteria. Low‐dose regimens (2–6.5 g/L) showed no significant difference compared to standard care (6–8.3 g/L) in PICD (reported in two studies) and renal dysfunction (reported in five studies). Other secondary outcomes, including hyponatremia, GI bleeding, HE, infections, and reaccumulating ascites, also showed no statistical differences. While these findings indicate potential for dose reduction, definitive confirmation via meta‐analysis was not feasible due to methodological heterogeneity. Available evidence suggests that reduced‐dose IV HA (≤ 6 g/L) is as effective and safe as standard‐dose HA (6–8 g/L) during LVP. However, the quality of the current evidence is limited by small sample sizes and suboptimal study designs, and well‐designed, adequately powered future studies are required to confirm these findings.

## Lay Summary

People with advanced liver disease can develop fluid build‐up in the abdomen (ascites), which is treated by draining the fluid and giving human albumin to prevent complications. The amount of albumin currently used is based on limited evidence, even though albumin is costly, scarce, and may cause side effects if overused. This review suggests that lower doses of albumin may be just as safe and effective as standard doses, but larger, high‐quality studies are needed to confirm this.

## Key Points

- A reduced dose of IV human albumin (2–6.5 g/L) during LVP appears to have similar safety and efficacy compared with standard dosing, though the level of evidence is low.
- Current studies are restricted by methodological limitations, heterogeneous dosing regimens, and reliance on surrogate endpoints like PICD.
- A well‐designed, adequately powered non‐inferiority trial is needed to confirm these findings.

**Abbreviations**
CSPH: clinically significant portal hypertension
HA: human albumin
HE: hepatic encephalopathy
HRS‐AKI: hepatorenal syndrome–acute kidney injury
LVP: large volume paracentesis
PICD: paracentesis‐induced circulatory dysfunction
rHA: recombinant human albumin
SBP: spontaneous bacterial peritonitis
VB: variceal bleeding

## 1 Introduction

Liver cirrhosis is a major global health issue, with prevalence rising to 25.3 per 100 000 in 2019 [1]. It results from chronic liver injury leading to fibrosis and ultimately advanced chronic liver disease. Common causes include obesity, alcohol use, viral infections, autoimmune and cholestatic diseases, and iron or copper overload [2]. Cirrhosis has two stages, compensated and decompensated, distinguished by the absence or presence of clinically significant portal hypertension (CSPH) [3]. CSPH, defined as a hepatic venous pressure gradient ≥ 10 mmHg, marks higher risk for decompensation events such as (refractory) ascites, spontaneous bacterial peritonitis (SBP), hepatorenal syndrome (HRS), variceal bleeding (VB), and hepatic encephalopathy (HE) [4].

Ascites is the most common decompensating event in cirrhosis, occurring in 5%–10% of patients annually [5]. A Dutch multicenter study found that 62% presented with ascites as the first decompensation [6]. Its presence marks transition to decompensated cirrhosis, with a three‐year mortality of ~50%, and < 50% one‐year survival in refractory cases. Main factors contributing to increased mortality risk are spontaneous bacterial peritonitis (SBP) and hepatorenal syndrome (HRS) [7]. The pathophysiology of ascites centers on portal hypertension as this causes accumulation of vasodilators such as nitric oxide, triggering RAAS activation contributing to renal impairment. As hydrostatic pressure and vascular stasis increase, fluid shifts from the liver and mesenteric vessels into the peritoneal cavity, leading to ascitic fluid accumulation [8].

Large‐volume paracentesis (LVP), arbitrarily defined as drainage of more than 5 L of ascitic fluid via a temporary transabdominal catheter, is used for therapy‐refractory or intractable ascites and to achieve rapid relief in patients with grade III (severe) ascites with marked abdominal distension [9, 10].

Paracentesis‐induced circulatory dysfunction (PICD) defined as a > 50% rise in plasma renin activity to > 4 ng/mL/h 6 days after LVP is a feared complication of LVP [11]. It results from splanchnic vasodilation and reduced systemic resistance, triggering neurohumoral activation and sodium and water retention. Clinically, PICD promotes rapid ascites recurrence, hyponatremia, hepatic encephalopathy, and hepatorenal syndrome‐associated AKI (HRS‐AKI) [11]. To mitigate these complications, intravenous (IV) human albumin (HA) is administered during LVP, which helps reduce the development of PICD from up to 80% to about 15% [12]. Guidelines recommend its use when more than 5 L of ascites is removed, as this reduces the risk of PICD and related decompensation [13, 14]. However, excessive administration can result in acute volume overload and pulmonary edema [15, 16]. Moreover, HA production is labor‐intensive and resource‐demanding since it is derived from human plasma, making it one of the most expensive plasma‐based products [17].

We aim to systematically review studies comparing low‐dose versus standard‐dose IV HA during LVP, hypothesizing that reduced dosing is equally safe. This review will summarize current evidence to guide future research.

## 2 Methods

### 2.1 Study Design

We conducted a systematic review to evaluate the available evidence on the safety of IV HA compared with the standard recommended dose in the context of LVP. The review was performed in accordance with the Preferred Reporting Items for Systematic Reviews and Meta‐Analysis (PRISMA) (Table S6) [18]. A PROSPERO protocol was pre‐registered (CRD42024586029).

### 2.2 Search Strategy

The search strategy was developed in collaboration with a medical librarian at Amsterdam UMC (F.J.). The following databases were systematically searched up to September 2024: PubMed, EMBASE, Cochrane Library, CINAHL and Scopus. The search incorporated terms such as “liver cirrhosis”, “ascites”, “paracentesis”, “albumin” and “dosage” (Tables S1 and S2). To maximize sensitivity, treatment outcomes were deliberately excluded from the search string.

### 2.3 Ongoing Trials

The search was initially conducted in September 2024. The search was updated to capture studies published between September 9, 2024, and January 31, 2025. The additional search was conducted in ClinicalTrials.gov and the World Health Organization International Clinical Trials Registry Platform (WHO‐ICTRP) to identify ongoing trials comparing HA dosages low versus at the time of LVP [19]. We combined the search terms “albumin” and “cirrhosis”. The obtained studies were screened on title, inclusion criteria and intervention. We reported the main study site, protocol publication year, recruitment and publication status.

### 2.4 Study Selection

**FIGURE 1:**
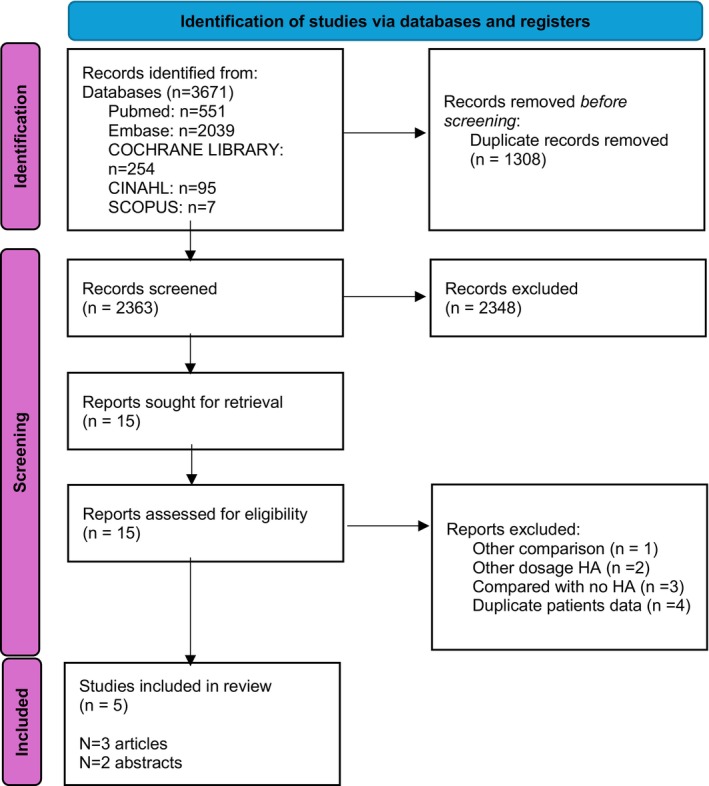
Flowchart of initial study selection. PRISMA 2020 flow diagram for new systematic reviews which included searches of databases and registers only.

Two investigators (R.H. and M.L.) independently screened titles and abstracts of identified studies with Rayyan, an application designed for study selection for systematic reviews [20]. Discrepancies were resolved through discussion with a third reviewer (S.B.Studies) comparing a low dose HA strategy (≤ 6 g HA per litre removed ascites) with the standard care dose HA strategy (> 6 g HA per litre removed ascites) were included for full text analysis. We used the Prisma flowchart diagram 2020 to present these results (Figure 1).

### 2.5 Eligibility Criteria

We included studies if all of the following items were met: (a) study in humans (≥ 18 years), (b) LVP because of decompensated liver cirrhosis, (c) comparison between a reduced (≤ 6 g HA administered per litre drained ascites or total amount of administered HA ≤ 20 g) with standard care (> 6 g HA administered per litre drained ascites or total amount of administered HA > 20 g). We excluded case reports and studies not published in English or Dutch.

### 2.6 Data Extraction

We extracted the following data from the included studies: author, study design, year of publication, country of origin, number of included patients, age, gender, aetiology of cirrhosis, Child‐Pugh score, Model for End‐Stage Liver Disease (MELD)‐score, total amount of administered HA, reported outcome, follow‐up duration and exclusion criteria. If the HA dosage was not reported in g/L, we estimated the HA dose by dividing [total amount of administered HA] by [drained ascites volume]. We extracted data on the following outcomes: PICD, AKI, hyponatremia, gastrointestinal (GI) bleeding including VBHE, refractory ascites, infections including SBP, transjugular intrahepatic portosystemic shunt (TIPS)‐free survival, transplant‐free survival, liver‐related mortality and all‐cause mortality.

### 2.7 Quality Assessment

We used the Cochrane Risk of Bias tool for the included Randomized Controlled Trials (Table S3) [21]. Additionally, the Cochrane Checklist was used to assess the methodological quality (validity) of RCTs (Table S4) [22]. The separate elements of these tools could score a study as: “low risk of bias”, “high risk of bias”, or “unclear risk of bias” if the element is not described. For cohort studies we used the Newcastle Ottawa Scale. This tool uses “stars” to rate the individual studies (Table S5) [23]. The data will be summarized in tables. We refrained from formal quality assessment for included abstracts.

### 2.8 Statistical Analysis

Continuous variables are expressed as mean with standard deviation or median with interquartile range, depending on the distribution of the data. Data was divided into two categories: between “low dose HA” (≤ 6 g/L or total amount ≤ 20 g) and “standard dose HA” (> 6 g/L or total amount > 20 g). Proportional change of individual outcomes between intervention (low HA dose) and control (standard HA dose). This data will be summarized in tables.

## 3 Results

### 3.1 Study Selection

Our search returned 3671 hits (Figure 1). We excluded *n* = 2348 articles by title and abstract screening. A full‐text assessment was conducted for the 15 remaining articles. Ten articles were excluded: two articles were excluded because the administered dose of HA did not meet our inclusion criteria (*n* = 1: intervention and control group received a similar HA dose; *n* = 1 HA dosage 10.3 g/L vs. 13.7 g/L). Four articles were excluded because the intervention used was either “no HA” or an alternative colloid or intravenous fluid (*n* = 3: dosage HA compared to no HA; *n* = 1 HA combined with spironolactone compared to HA only). Four articles were excluded due to data duplication, as they reported patient data already present in an included study. Finally, three full‐text articles and two abstracts were included for this systematic review.

### 3.2 Ongoing Trials

**FIGURE 2:**
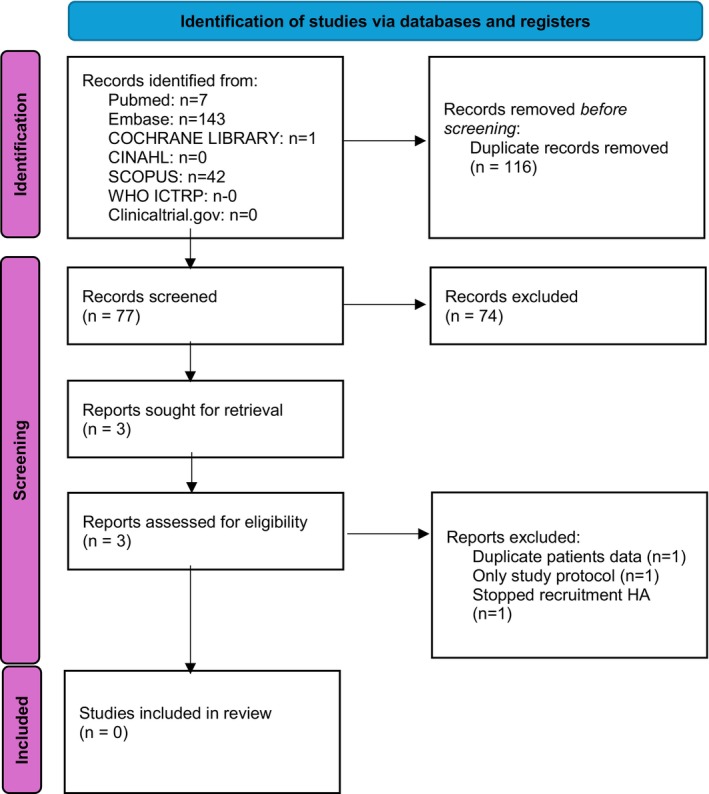
Flowchart of updated study selection. PRISMA 2020 flow diagram for new systematic reviews which included searches of databases and registers only.

Expanding the search to January 31 2025 did not reveal any additional hits (Figure 2). Only three trials met our inclusion criteria. One of these was a duplicate (NCT00428506) of the published study of Alessandria et al. [12]. The study coordinator (Turin, Italy) of another trial (EUCTR2007‐001733‐34‐IT) published their protocol back in 2007, the current status of this study is unknown despite our effort to contact the study coordinator The last identified trial dates back to 2010 (EUCTR2010‐019783‐37‐CZ) and stopped patient recruitment. This study was initiated from the Trebic hospital, located in the Czech republic. We could not contact the study coordinator. To conclude, there is no active study registered investigating the efficacy and safety of a reduced HA dose at the time of LVP.

### 3.3 Baseline Characteristics

A total of five studies were included: three randomized controlled trials (RCTs), one case series, and one retrospective cohort study (Table 1). All studies were relatively recent, with the earliest published in 2011. Sample sizes ranged from 50 to 200 participants, yielding a combined total of 527 patients. The administered HA dose varied between studies, most commonly being 4 g/L in the intervention group and 8 g/L in the comparison group. Across studies, the majority of participants were male, and most study patients were between 50 and 55 years of age. The mean Child‐Pugh score ranged from 7 to 10, and the most common etiologies of cirrhosis were alcohol‐related liver disease and viral hepatitis.

**TABLE 1:** Study characteristics.

	Alessandria [15]	Khalid [24]	Al Sebaey [23]	Anderson [25]	Naidu [26]
Study design	RCT	Case series	RCT	Retrospective cohort	RCT
Population	Intervention group, *n* = 35	Intervention group, *n* = 110	Intervention group, *n* = 25	Intervention group, *n* = 100	Intervention group, *n* = 33
	Comparison group, *n* = 35	Comparison group, *n* = 31	Comparison group, *n* = 25	Comparison group, *n* = 100	Comparison group, *n* = 33
Publication year	2011	2011	2013	2018	2024
Intervention	4 g/L HA	3.7 g/L HA	2 g/L HA	6.5 g/L HA	4 g/L HA
Comparison	8 g/L HA	5.9 g/L HA	6 g/L HA	8.3 g/L HA	8 g/L HA
Male	*n* = 57 (81.4%)	—	*n* = 69 (55.2%)	*n* = 145 (74.5%)	*n* = 56 (84.8%)
Mean age (years)	Intervention group, 55 ± 12	Intervention group, 54 ± 11	Intervention group 49.08 ± 7.43	Intervention group 55	Intervention group, 52,24 ± 11,03
	Comparison group 57 ± 11 *p* = 0.8	Comparison group 53 ± 10 *p* = N/A	Comparison group 51.60 ± 6.84 *p* > 0.05	Comparison group 55 *p* = 0.96	Comparison group 54.83 ± 13.08 *p* = 0.39
Mean Child‐Pugh score	Intervention group 10 ± 2	Intervention group 10–15 at 70%	Intervention group 9.88 ± 1.17	Intervention group 7–9 at 64% and > 10 at 36%	Intervention group 7–9, 33.3% and 10–15, 66.7%
	Comparison group 10 ± 2 *p* = 0.9	Comparison group 10–15 at 70% *p* = N/A	Comparison group 9.88 ± 1.56 *p* > 0.05	Comparison group 7–9 at 61% and > 10 at 39% *p* = 0.66	Comparison group 7–9 42.2% and 10–15 57.6% *p* = 0,447
Aetiology	Intervention group 34% AA	Intervention group 70% HCV	Intervention group 84% HCV, 8% HBV, 4% Budd‐Chiari syndrome, 4% cryptogenic	Intervention group 40% AA, 17% HCV, 23% AA and HCV, 13% NASH, 7% other	Intervention group 63.3% AA, 15.2% HCV/HBV
	Comparison group 40% AA *p* = 0.8	Comparison group 55% HCV *p* = N/A	Comparison group 84% HCV, 8% HBV, 8% Budd‐Chiari syndrome *p* > 0.05	Comparison group 57% AA, 18% HCV, 12% AA and HCV, 9% NASH, 4% other *p* = 0.09	Comparison group 78.8% AA, 6.1% HCV/HBV *p* > 0.05
Primary outcome	PICD (6 days after LVP)	Serum creatinine, serum sodium (1 week after LVP)	PICD (6 days after LVP)	Amount of albumin used after LVP	Renal dysfunction, hyponatremia, ascites reaccumulation (6 days after LVP)
Secondary outcome	Renal impairment, hyponatremia, GI bleeding, hepatic encephalopathy, infections (6 days after LVP)	N/A	Renal impairment and hyponatremia (6 days after LVP)	Hyponatremia, renal impairment, hypotension	N/A
Follow up	6 months	N/A	N/A	N/A	N/A
Exclusion criteria	Patients with MHCC, PVT, bacterial infection, ongoing or recent bleeding, CF, creat > 2 mg/dL, IRD, vasoactive drugs, recent use of PE	Patients with SBP, creat > 1.5 mg/dL, hepatoma, coexisting malignancy, LVP < 5 L	Patients with arterial hypertension, history of CD, CF, RD, HE, elevated creat > 1.5 mg/dl, and GI‐bleeding < 7 days before the study	Patients with DP, other albumin orders outside the orderset	Patients with SBP, MA, CF, AKI, PVT, VB 7 days before LVP, recent use of PE

Abbreviations: AA, alcohol abuse; CD, cardiac disease; CF, cardiac failure; Creat, Creatinine; DP, diagnostic paracentesis; GI, gastro‐intestinal; HA, human albumin; HBV, hepatitis B; HCV, hepatitis C; HE, hepatic encephalopathy; IRD, intrinsic renal disease; LVP, large volume paracentesis; MA, malignant ascites; MHCC, Multinodulatr hepato‐ cellular carcinoma; N/A, not available; NASH, non alcoholic fatty liver disease; PE, plasma expanders; PICD, paracentesis‐induced circulatory dysfunction; PVT, portal vein thrombosis; RD, respiratory disease; SBP, spontaneous bacterial peritonitis; VB, variceal bleeding.

### 3.4 Renal Impairment

All study‐related outcomes are reported in Table 2. In the case of renal impairment, this outcome was defined as a > 50% increase in serum creatinine from baseline with a minimum level of 1.5 mg/dL (132 μmol/L). None of the studies demonstrated a significant difference in the incidence of renal impairment between the intervention and comparison groups.

**TABLE 2:** Study results.

	PICD	Renal impairement	Hyponatremia	GI‐bleeding	Encephalopathy	Infections	Ascites reaccumulation
Alessandria [15]	Intervention 14.2% (*n* = 5)	Intervention 0%	Intervention 9% (*n* = 3)	Intervention 0%	Intervention 0%	Intervention 0%	N/A
	Comparison 20% (*n* = 7) *p* = NS	Comparison 0% *p* = NS	Comparison 6% (*n* = 2) *p* = NS	Comparison 0% *p* = NS	Comparison 0% *p* = NS	Comparison 0% *p* = NS	N/A
Khalid [24]	N/A	Intervention serum create 1.07 ± 0.03 mg/dL	Intervention serum natrium 129.62 ± 4.1 mmol/L	N/A	N/A	N/A	N/A
	N/A	Comparison serum creat 1.41 ± 0.17 mg/dL (mean ± SD) *p* = 0.35	Comparison serum natrium 128.7 ± 6.0 mmol/L (mean ± SD) *p* = 0.14	N/A	N/A	N/A	N/A
Al Sebaey [23]	Intervention 12% (*n* = 3)	Intervention serum creat Δ0.10 ± 0.21 mg/dL	Intervention serum sodium Δ 3.32 ± 1.14 mmol/L	N/A	N/A	N/A	N/A
	Comparison 12% (*n* = 3) *p* > 0.05	Comparison serum creat Δ0,06 ± 0.29 mg/dL (mean ± SD) *p* > 0.05	Comparison serum sodium Δ2,72 ± 1.43 mmol/L (mean ± SD) *p* > 0.05	N/A	N/A	N/A	N/A
Anderson [25]	N/A	Intervention 11.5%	Intervention 6.6%	N/A	N/A	N/A	N/A
	N/A	Comparison 11.3% *p* = 0.97	Comparison 1.6% *p* = 0.21	N/A	N/A	N/A	N/A
Naidu [26]	N/A	Intervention 24.2% (*n* = 8)	Intervention 33.3% (*n* = 11)	N/A	N/A	N/A	Intervention 33.3%
	N/A	Comparison 18.2% (*n* = 6) *p* = 0.547	Comparison 27.3% (*n* = 9) *p* = 0.592	N/A	N/A	N/A	Comparison 24.2% *p* = 0.516

Abbreviations: N/A, not available; NS, not significant.

### 3.5 Hyponatremia

Hyponatremia was defined as a decrease in serum sodium > 5 mmol/L to levels < 130 mmol/L on the sixth day post‐paracentesis. Patients with baseline sodium < 130 mmol/L who experienced a reduction > 5 mmol/L were also considered hyponatremic. Across all studies, no significant differences were observed between groups.

### 3.6 PICD

Two studies, by Alessandria et al. and Alsebaey et al., reported on the incidence of PICD, assessed 6 days post‐LVP [25]. PICD was defined as a > 50% increase in plasma renin activity (PRA) compared with pretreatment values. In both studies, no significant difference in PICD incidence was found between dosing groups.

### 3.7 GI‐Bleeding, HE, Infections and Reaccumulating Ascites

Only the study by Alessandria et al. reported outcomes for gastrointestinal bleeding, hepatic encephalopathy (HE), and infections, with no significant differences between the intervention and comparison groups [12]. For recurrent ascites, the study by Naidu et al. reported a recurrence rate of 33.3% in the intervention group versus 24.2% in the comparison group; however, this difference was not statistically significant [24].

### 3.8 Quality Assessment

The study of Alessandria et al. is a RCT that used sealed opaque envelopes for randomization, which gave a low risk of bias (Table 3) [12]. The trial had an open‐label design; because of this, outcome assessment was not blinded and resulted in a high risk of bias. None were lost to follow‐up, and all participants were analysed according to their original group allocation (intention‐to‐treat). A study protocol is available online and was consistent with the published article. No conflicts of interest were reported, resulting in a low risk in selective reporting and influence of sponsoring. Overall, most elements were assessed with a low risk of bias and two elements with a high risk of bias. Following the Cochrane RoB‐tool, the overall quality is poor (Table S3).

**TABLE 3:** Quality assessment of included randomized clinical trials.

	D1	D2	D3	D4	D5	Overall bias
Alessandria 2011, Italy [15]	NA	Low	Low	Low	High	High
Al Sebaey 2013, Egypt [23]	Some concerns	High	High	Low	High	High

*Note:* The study of Alessandria et al. scored on D2‐D4 a low risk of bias because of the kind of randomization, the clear results, no conflicts of interest and 100% follow up. A high risk of bias was found in the fact that it is an open label trial. The study of Al Sebaey et al. scored high risk of bias because it did not have a published study protocol and was unclear in their randomization and blinding procedures.

Abbreviations: D1, randomization process; D2, deviations from intended interventions; D3, missing outcome data; D4, measurement of the outcome; D5, selection of the reported result.

The study of Alsebaey et al. is a RCT that did randomize; however, randomization and blinding procedures for patients, practitioners, and reviewers were not specified, which was classified as a high risk of bias (Table 3) [25]. This study reported no loss to follow‐up and study participants were analysed using an intention‐to‐treat analysis, resulting in a low risk of bias. Absence of a published study protocol resulted in a high risk of bias. Following the Cochrane RoB‐tool the overall quality is poor (Table S3).

The study of Anderson et al. is a retrospective cohort study. Although baseline Child‐Pugh score was comparable between groups, the baseline incidence of chronic kidney disease between intervention and control groups was significantly different, resulting in a one star rating (low risk of bias) following the New‐castle‐Ottawa scales (Table 4) [26]. The intervention and control cohorts were drawn from the same community representing a two star rating (low risk of bias). Study outcomes were clearly described and assessed with SPSS, record linkage, resulting in two stars as well. The study reported missing data for primary outcome measurements (serum sodium and serum creatinine data were unavailable for 38.5% of patients, while systolic blood pressure data were missing for 8.5% respectively). This is categorized as no star. The follow up was long enough, assessed with one star. Overall, it scored three stars in the selection domain, one star in the comparability domain and two stars in the outcome domain, resulting in an overall moderate quality.

**TABLE 4:** Quality assessment of cohort study.

	Representativeness	Ascertainment of exposure	Non exposed cohort selection	Outcome Not present before start	Follow up	Outcome assessment	Comparability groups
Anderson 2018, USA	*	*	*		*	*	*

*Note:* The study of Anderson et al. is of moderate quality. The cohorts are representative, selected out of the same community and comparable on age and sex. The outcome was a secure report assessed with SPSS. The follow up was long enough but the rate was too low.

The studies of Khalid et al. and Naidu et al. were only available as abstracts, and therefore no formal critical appraisal was performed [24, 27].

## 4 Discussion

This systematic review provides an overview of the available evidence comparing the safety and efficacy of a low‐dose IV HA regimen versus the standard dose during LVP. Across all included trials, reduced‐dose albumin (2–6.5 g/L) was not associated with worse outcomes compared with standard dosing, suggesting that a reduced albumin dose may be as safe as the standard regimen. Notably, for endpoints including renal impairment, hyponatremia, PICD, gastrointestinal bleeding, HE, infections, and ascites reaccumulation, no significant differences were observed between groups. However, the quality of evidence is limited: a meta‐analysis was not feasible due to the limited number of RCTs and, more importantly, because of significant methodological limitations in the available studies. While these findings are not sufficient to draw firm conclusions or provide clinical recommendations, they do highlight an important gap in knowledge and underscore the need for well‐designed trials to determine optimal dosing strategies.

The relevance of this question is underscored by the substantial clinical and economic burden of HA use. HA is well‐established as the most effective agent for preventing LVP‐related complications, yet its high cost and global scarcity pose significant challenges. In the Netherlands, the incidence of cirrhosis rose from 48.8 per 100 000 adults in 2017 to 75.2 per 100 000 in 2021. Over the same period, hospital admissions increased to 2899 annually, while healthcare expenditures more than doubled, from €35 million to €78 million [28]. In addition, the expected expansion of HA indications, coupled with declining donor availability, presents a critical challenge: future albumin shortages are anticipated, highlighting the urgent need for responsible use and optimized dosing strategies [29]. This is particularly relevant given the high cost of HA in the Netherlands, where a single 100 mL vial containing 200 mg/mL (20 g total) costs €104.64 [30]. Applying the standard 8 g/L HA regimen to all estimated 10 350 LVPs performed annually in the Netherlands, based on data from more than 20 hospitals in 2024, would cost approximately €4.33 million in albumin alone. Given that 62% of patients present with ascites as their first decompensating event, reducing the HA dose could generate substantial direct savings on albumin, as well as further reductions in overall healthcare costs, including hospital resources and management of LVP‐related complications [13, 31, 32]. Globally, the HA market is projected to expand markedly, from $5 billion in 2021 to $10 billion by 2032 [33]. Therapeutic applications currently account for 47.3% of total HA use, and the prevalence of chronic diseases is expected to rise by 57% worldwide, further driving demand [33].

A potential solution for the high costs and expanding clinical use of albumin is the development of recombinant human albumin (rHA) as an alternative to plasma‐derived products. rHA is produced in genetically engineered organisms, including yeast, bacteria, mammalian cell cultures, silkworm cocoons, and transgenic plants or animals. Despite its promise, the production process remains challenging, particularly because it requires extremely high purity to minimize the risk of immunogenic host‐cell contaminants [34]. While rHA offers potential for greater scalability and reduced dependence on donor plasma, it should currently be considered a complementary rather than a replacement therapy.

Several studies have evaluated the cost‐effectiveness of HA. In Indonesia with a resource‐constrained healthcare setting, HA was found to be cost‐effective in preventing paracentesis‐induced circulatory dysfunction (PICD) [35]. Similarly, Runken et al. evaluated the use of HA for decompensated cirrhosis in Germany, Italy, and Spain and determined that it is more effective as well as cheaper than alternatives such as saline, gelatin, or no plasma expander. The benefits result from the fact that HA prevents complications that would otherwise require additional interventions, longer hospital stays, and greater healthcare resources. In this analysis, the absence of fluid replacement was associated with the poorest outcomes and lowest quality‐adjusted life years (QALYs) [36]. In line with these findings, several systematic reviews and meta‐analyses comparing HA with alternative plasma expanders consistently concluded that HA remains the most effective option compared to other plasma expanders [13, 37, 38].

Alternative strategies, including the use of midodrine, have also been explored and may be similarly effective or potentially superior to HA in preventing PICD [11, 39]. However, subsequent studies have reported less favourable outcomes with midodrine compared with HA [40]. Practical limitations further restrict the use of midodrine, as it is not reimbursed in all European countries, including the Netherlands [41]. Moreover, HA possesses beneficial immunomodulatory properties that midodrine lacks. These considerations suggest that while alternative agents may have potential, albumin remains the preferred option in most clinical contexts.

Despite its well‐established benefits, the minimal effective dose of HA remains unclear. The current recommendation of 6–8 g was empirically derived in the 1980s by estimating HA losses during LVP [13], rather than being determined through dose–response trials leading to practice variation [42, 43]. This dose appeared to reduce complications such as AKI and hyponatremia, but its full effects and potential downsides were never comprehensively studied [13]. Noteworthy, the risk of PICD (and risk of decompensation) is absent in patients in whom 5 L or less ascites is drained during a single session [14].

Earlier reviews have suggested that lower doses might preserve efficacy while reducing costs. However, despite longstanding interest, no large randomized controlled trials have been conducted, and guideline recommendations remain unchanged.

The present review was unable to provide sufficient evidence to support changes to current practice, but several important lessons emerge.

First, the choice of primary outcome is crucial. PICD (based on PRA) has historically been the preferred primary outcome measure for comparing the efficacy and safety of different colloid/fluid replacement strategies during LVP. However, using PICD as the primary outcome in trials presents several disadvantages. First, the original studies measuring PRA date back to the 1980s. More recent investigations have shown: (a) no significant difference in PRA levels before LVP vs. 1, 2, or 6 days after LVP [44], and (b) that PICD is a common finding following LVP, even when 6–8 g/L of IV HA is administered [45]. Furthermore, a recent Cochrane meta‐analysis observed no correlation between PICD and renal failure or mortality [46]. A retrospective study of patients receiving standard‐care IV HA at the time of LVP found no significant differences in systemic hemodynamics, renal function, or survival between those who did or did not develop PICD [45].

Second, the timing of outcome assessment warrants reconsideration. PICD is typically assessed 6 days after LVP, and most studies assessed endpoints at 6 days post‐LVP. However, patients often undergo repeated LVP procedures over months or years, and any cumulative harmful effect of reducing HA doses might be missed by such a short observation period. Therefore, outcomes other than PICD should be used to compare the efficacy and safety of different HA doses during LVP [47]. These outcomes should reflect long‐term prognosis, such as the development of new decompensating events, providing a more meaningful assessment of safety [48].

While several studies have investigated albumin use during large‐volume paracentesis, most have focused on alternative or adjunctive therapies and have accepted the standard albumin dose as a given [11, 39]. Moreover, prior research has frequently relied on PICD as a primary endpoint, despite its limited association with clinically relevant outcomes [49]. The present review differs by specifically examining whether reduced‐dose albumin is sufficient and by emphasizing the need for future studies based on meaningful long‐term clinical endpoints.

Future studies should investigate whether reduced‐dose HA can be safely implemented in clinical practice. Since the current standard of care is based on expert opinion, designing future studies on HA dosing requires careful consideration [50, 51]. To address this, we conducted a three‐round Delphi study involving 23 international experts [52]. The process informed an international position statement on HA use in cirrhosis and highlighted the limited evidence regarding the minimum effective dose of HA for preventing PICD [53]. The panel reached consensus on key design elements for a non‐inferiority trial. They recommended a composite primary endpoint at 52 weeks, including refractory ascites, SBP, HRS‐AKI, VB, and HE, with a non‐inferiority margin of 10%–12% based on an anticipated 65% incidence rate [5]. Assuming a 10% margin, one‐sided alpha of 5%, 80% power, and 10% drop‐out, 634 patients would be required to establish non‐inferiority of low‐dose IV HA. The panel also recommended excluding patients with active AKI, hemodynamic instability, systemic infection, VB, and acute‐on‐chronic liver failure, and advised stratification by prior history of AKI to enable nuanced subgroup analyses and strengthen the study's conclusions.

This study has several strengths. First, it addresses an underexplored but highly relevant aspect of human HA use, a product that is both costly and scarce. It provides a structured overview of all available data on reduced dosing during LVP and places these findings in the context of earlier key publications. In addition, the discussion underscores the clinical and economic relevance of this question and outlines clear recommendations for future research.

This study also has limitations. Most importantly, the number of available RCTs is limited, which precluded the conduct of a meta‐analysis. The included studies were heterogenous with respect to their HA dosing regimens, and the power of the studies was limited. Finally, the trial by Khalid et al. only reported serum creatinine and serum sodium, which we therefore adopted as indicators of renal impairment and hyponatremia in our review, although these reflect indirect measures [27].

In conclusion, although the existing literature suggests that low‐dose IV HA may be as safe as standard‐care IV HA during LVP, the limited number of randomized controlled trials, varying dosing regimens, and methodological constraints prevent a definitive conclusion on its non‐inferiority. Given the significant clinical and economic burden of liver cirrhosis, as well as the potential cost savings associated with reducing HA usage, a well‐designed and adequately powered non‐inferiority trial is needed to determine whether a low IV HA dose truly maintains equivalent efficacy and safety.

## Author Contributions

M.A.L., K.K.I.M.B., R.H. and J.P.H.D. contributed to concept and design. R.H. and M.A.L. performed the systematic literature search. R.H. and M.A.L. performed analyses. All authors contributed to interpretation of results. All authors contributed to writing of the article.

## Funding

The authors have nothing to report.

## Ethics Statement

Ethical approval was not required for this study as it is a systematic review based exclusively on data from previously published studies.

## Consent

This study is a systematic review of previously published literature and does not involve individual patients or the collection of new patient data.

## Conflicts of Interest

The authors declare no conflicts of interest.

## Supporting information


**Table S1:** Initial search.
**Table S2:** Updated search.
**Table S3:** Risk of bias.
**Table S4:** Cochrane checklist RCT's.
**Table S5:** Quality assessment.
**Table S6:** PRISMA 2020 checklist.

## Data Availability

The data that support the findings of this study are available from the corresponding author upon reasonable request.
