# Supplementary material for: Low‐Dose vs. Standard Care Iv Human Albumin During Large‐Volume Paracentesis in Patients With Liver Cirrhosis: A Systematic Review
Source: Liver Int. 2026 Mar 26;46(5):e70621. doi: 10.1111/liv.70621 (PMC13022514; doi:10.1111/liv.70621)
Supplement: Supplementary file 1 — Table S1: Initial search. Table S2: Updated search. Table S3: Risk of bias. Table S4: Cochrane checklist RCT's. Table S5: Quality assessment. Table S6: PRISMA 2020 checklist. [file LIV-46-0-s001.docx]

## **Supplementary**

Title

Low-dose vs. standard care iv human albumin during large-volume paracentesis in patients with liver cirrhosis: a systematic review

Authors

Kimberly K.I.M. Bot

Roebi Heus

Joost PH Drenth

Marten A Lantinga

Table of contents

1. Supplementary table 1: Initial search
2. Supplementary table 2: Updated search
3. Supplementary table 3: Risk of bias
4. Supplementary table 4: Cochrane checklist RCT’s
5. Supplementary table 5: Quality assessment
6. Supplementary table 6: PRISMA 2020 checklist
7. Supplementary references

### Supplementary table 1. Initial search

**PUBMED**

**551 RESULTS**

**("Liver Cirrhosis"[Mesh] OR cirrho*[tiab])**

**AND**

**("Ascites"[Mesh] OR ascit*[tiab] OR ascet*[tiab])**

**AND**

**("Paracentesis"[Mesh] OR "Drainage"[Mesh] OR paracent*[tiab] OR drain*[tiab] OR surgical punctur*[tiab])**

**AND**

**(("Albumins"[Mesh] OR "Plasma Substitutes"[Mesh] OR "Diuretics"[Mesh] OR "Colloids"[Mesh] OR "Dextrans"[Mesh] OR "Polygeline"[Mesh] OR "Ascitic Fluid"[Mesh] OR albumin*[tiab] OR diuretic*[tiab] OR colloid*[tiab] OR dextran*[tiab] OR polygeline*[tiab] OR reinfusion*[tiab] OR plasma substitute*[tiab] OR ascites fluid*[tiab] OR haemaccel[tiab] OR haemacel[tiab] OR hemaccel[tiab] OR hydroxy starch[tiab]) OR ((volume[tiab] OR plasma[tiab]) AND expan*[tiab]))**

**AND**

**("administration and dosage" [Subheading] OR "Drug Therapy"[Mesh] OR "drug therapy" [Subheading] OR dose*[tiab] OR dosage*[tiab] OR dosing*[tiab] OR standard[tiab] OR low[tiab] OR lower*[tiab] OR half[tiab] OR administrat*[tiab] OR manag*[tiab] OR suppletion*[tiab])**

**EMBASE (OVID):**

**Database(s): Embase Classic+Embase 1947 to 2024 September 06
 Search Strategy:**

| **#** | **Searches** | **Results** |
| --- | --- | --- |
| **1** | **exp liver cirrhosis/** | **226502** |
| **2** | **cirrho*.ti,ab,kf.** | **214003** |
| **3** | **1 or 2** | **274724** |
| **4** | **exp ascites/** | **76386** |
| **5** | **(ascit* or ascet*).ti,ab,kf.** | **88507** |
| **6** | **4 or 5** | **112897** |
| **7** | **exp paracentesis/ or surgical drainage/ or exp abdominal drainage/** | **127051** |
| **8** | **(paracent* or drain* or surgical punctur*).ti,ab,kf.** | **265286** |
| **9** | **7 or 8** | **364408** |
| **10** | **exp albumin/ or exp plasma substitute/ or exp diuretic agent/ or exp diuretic therapy/ or exp colloid/ or exp dextran/ or exp ascites fluid/ or polygeline/** | **827180** |
| **11** | **(albumin* or diuretic* or colloid* or dextran* or polygeline* or reinfusion* or plasma substitute* or ascites fluid* or haemaccel or haemacel or hemaccel or hydroxy starch*).ti,ab,kf.** | **510879** |
| **12** | **((volume* or plasma*) and expan*).ti,ab,kf.** | **71557** |
| **13** | **10 or 11 or 12** | **1157207** |
| **14** | **exp drug administration/ or exp drug therapy/ or exp drug dose/ or exp dose/ or (drug administration or intravenous drug administration).fs.** | **5634073** |
| **15** | **(dose* or dosage* or dosing* or standard or low or lower* or half or administrat* or manag* or suppletion*).ti,ab,kf.** | **13319041** |
| **16** | **14 or 15** | **16188659** |
| **17** | **3 and 6 and 9 and 13 and 16** | **2039** |

**COCHRANE LIBRARY (Wiley):**

[**Cochrane Central Register of Controlled Trials**](https://www.cochranelibrary.com/)

**Issue 8 of 12, August 2024**

[**Cochrane Database of Systematic Reviews**](https://www.cochranelibrary.com/)

**Issue 9 of 12, September 2024**

**ID Search Hits**

**#1 (liver* NEAR3 cirrh*):ti,ab,kw 70487**

**#2 (ascit* or ascet*):ti,ab,kw 3554**

**#3 (paracent* or drain* or surgical punctur*):ti,ab,kw 17798**

**#4 (albumin* or diuretic* or colloid* or dextran* or polygeline* or reinfusion* or plasma substitute* or ascites fluid* or haemaccel or haemacel or hemaccel or hydroxy starch*):ti,ab,kw 36553**

**#5 ((volume* OR plasma*) AND expan*):ti,ab,kw 4269**

**#6 #4 or #5 40267**

**#7 (dose* or dosage* or dosing* or standard or low or lower* or half or administrat* or manag* or suppletion* or plasma expander* or volume expander*):ti,ab,kw 1192410**

**#8 #1 and #2 and #3 and #6 and #7 in Cochrane Reviews, Trials 254**

**CINAHL (EBSCO):**

**95 results**

**(MH "Liver Cirrhosis+") OR TI cirrho* OR AB cirrho***

**AND**

**(MH "Ascites") OR TI ( ascit* or ascet* ) OR AB ( ascit* or ascet* )**

**AND**

**( (MH "Paracentesis+") OR (MH "Drainage+") ) OR TI ( paracent* or drain* ) OR AB ( paracent* or drain* )**

**AND**

**(MH "Albumins+") OR ( (MH "Plasma Substitutes+") OR (MH "Colloids+") ) OR (MH "Diuretics+") OR (MH "Dextrans") OR TI ( albumin* or diuretic* or colloid* or dextran* or polygeline* or reinfusion* or plasma substitute* or ascites fluid* or haemaccel or haemacel or hemaccel or hydroxy starch* or volume expander* or plasma expander* ) OR AB ( albumin* or diuretic* or colloid* or dextran* or polygeline* or reinfusion* or plasma substitute* or ascites fluid* or haemaccel or haemacel or hemaccel or hydroxy starch* or volume expander* or plasma expander* )**

**SCOPUS (Elsevier):**

**732 results**

**(TITLE-ABS-KEY("liver cirrhosis" OR cirrho*) AND TITLE-ABS-KEY(ascites OR ascetic) AND TITLE-ABS-KEY(paracentesis* OR drain*) AND TITLE-ABS-KEY(albumin* OR diuretic* OR colloid* OR dextran* OR polygeline* OR reinfusion* OR "plasma substitute*" OR "ascites fluid*" OR haemaccel OR haemacel OR hemaccel OR "hydroxy starch*") AND TITLE-ABS-KEY(" drug dos*" OR "low dos*" OR "lower dos*" OR "half dos*" OR administrat* OR manag* OR suppletion*))**

### Supplementary table 2. Updated search

Search: Faridi Jamaludin, Amsterdam UMC location University of Amsterdam, Medical Library AMC, Meibergdreef 9, Amsterdam, The Netherlands

**Search UPDATE 9-9-2024 - 31-1-2025**

**31-1-2025**:

| Databases: | Before deduplication | After deduplication* | After deduplication with references of the search 9-9-2024 |
| --- | --- | --- | --- |
| PubMed  Embase (Ovid)  Cochrane Library (Wiley): CDRS en CENTRAL  CINAHL (Ebsco)  SCOPUS  Clinicaltrial.gov  WHO ICTRP search portal |  |  |  |
| Total | 193 | 161 | 77 |

*Lobbestael, G. (2023). DedupEndNote (Version 1.0.0) [Computer software]. https://github.com/globbestael/DedupEndNote

PUBMED 7 results

("Liver Cirrhosis"[Mesh] OR cirrho*[tiab])

AND

("Ascites"[Mesh] OR ascit*[tiab] OR ascet*[tiab])

AND

("Paracentesis"[Mesh] OR "Drainage"[Mesh] OR paracent*[tiab] OR drain*[tiab] OR surgical punctur*[tiab])

AND

(("Albumins"[Mesh] OR "Plasma Substitutes"[Mesh] OR "Diuretics"[Mesh] OR "Colloids"[Mesh] OR "Dextrans"[Mesh] OR "Polygeline"[Mesh] OR "Ascitic Fluid"[Mesh] OR albumin*[tiab] OR diuretic*[tiab] OR colloid*[tiab] OR dextran*[tiab] OR polygeline*[tiab] OR reinfusion*[tiab] OR plasma substitute*[tiab] OR ascites fluid*[tiab] OR haemaccel[tiab] OR haemacel[tiab] OR hemaccel[tiab] OR hydroxy starch[tiab]) OR ((volume[tiab] OR plasma[tiab]) AND expan*[tiab]))

AND

("administration and dosage" [Subheading] OR "Drug Therapy"[Mesh] OR "drug therapy" [Subheading] OR dose*[tiab] OR dosage*[tiab] OR dosing*[tiab] OR standard[tiab] OR low[tiab] OR lower*[tiab] OR half[tiab] OR administrat*[tiab] OR manag*[tiab] OR suppletion*[tiab])

AND

("2024/09/09"[Date - Publication] : "2025/01/31"[Date - Publication])

EMBASE (OVID):

Database(s): Embase Classic+Embase 1947 to 2025 January 30
Search Strategy:

| **#** | **Searches** | **Results** |
| --- | --- | --- |
| 1 | exp liver cirrhosis/ | 232911 |
| 2 | cirrho*.ti,ab,kf. | 219536 |
| 3 | 1 or 2 | 282686 |
| 4 | exp ascites/ | 78252 |
| 5 | (ascit* or ascet*).ti,ab,kf. | 90736 |
| 6 | 4 or 5 | 115625 |
| 7 | exp paracentesis/ or surgical drainage/ or exp abdominal drainage/ | 130101 |
| 8 | (paracent* or drain* or surgical punctur*).ti,ab,kf. | 269470 |
| 9 | 7 or 8 | 371027 |
| 10 | exp albumin/ or exp plasma substitute/ or exp diuretic agent/ or exp diuretic therapy/ or exp colloid/ or exp dextran/ or exp ascites fluid/ or polygeline/ | 843804 |
| 11 | (albumin* or diuretic* or colloid* or dextran* or polygeline* or reinfusion* or plasma substitute* or ascites fluid* or haemaccel or haemacel or hemaccel or hydroxy starch*).ti,ab,kf. | 519150 |
| 12 | ((volume* or plasma*) and expan*).ti,ab,kf. | 72649 |
| 13 | 10 or 11 or 12 | 1176995 |
| 14 | exp drug administration/ or exp drug therapy/ or exp drug dose/ or exp dose/ or (drug administration or intravenous drug administration).fs. | 5796456 |
| 15 | (dose* or dosage* or dosing* or standard or low or lower* or half or administrat* or manag* or suppletion*).ti,ab,kf. | 13503716 |
| 16 | 14 or 15 | 16450735 |
| 17 | 3 and 6 and 9 and 13 and 16 | 2099 |
| 18 | limit 17 to yr="2024 -Current" | 143 |

[*Cochrane* Central Register of Controlled Trials](https://www.cochranelibrary.com/)

Issue 12 of 12, December 2024

ID Search Hits

#1 (liver* NEAR/3 cirrh*):ti,ab,kw 9101

#2 (ascit* or ascet*):ti,ab,kw 3630

#3 (paracent* or drain* or surgical punctur*):ti,ab,kw 18295

#4 (albumin* or diuretic* or colloid* or dextran* or polygeline* or reinfusion* or plasma substitute* or ascites fluid* or haemaccel or haemacel or hemaccel or hydroxy starch*):ti,ab,kw 37273

#5 ((volume* OR plasma*) AND expan*):ti,ab,kw 4378

#6 #4 or #5 41089

#7 (dose* or dosage* or dosing* or standard or low or lower* or half or administrat* or manag* or suppletion* or plasma expander* or volume expander*):ti,ab,kw 1220794

#8 #1 and #2 and #3 and #6 and #7 with Cochrane Library publication date Between Sep 2024 and Jan 2025, in Cochrane Reviews, Trials 1

SCOPUS (Elsevier):

42 results

( TITLE-ABS-KEY ( "liver cirrhosis" OR cirrho* ) AND TITLE-ABS-KEY ( ascites OR ascetic ) AND TITLE-ABS-KEY ( paracentesis* OR drain* ) AND TITLE-ABS-KEY ( albumin* OR diuretic* OR colloid* OR dextran* OR polygeline* OR reinfusion* OR "plasma substitute*" OR "ascites fluid*" OR haemaccel OR haemacel OR hemaccel OR "hydroxy starch*" ) AND TITLE-ABS-KEY ( " drug dos*" OR "low dos*" OR "lower dos*" OR "half dos*" OR administrat* OR manag* OR suppletion* ) ) AND PUBYEAR > 2023 AND PUBYEAR < 2026

CINAHL (EBSCO): 0 new results:

(MH "Liver Cirrhosis+") OR TI cirrho* OR AB cirrho*

AND

(MH "Ascites") OR TI ( ascit* or ascet* ) OR AB ( ascit* or ascet* )

AND

( (MH "Paracentesis+") OR (MH "Drainage+") ) OR TI ( paracent* or drain* ) OR AB ( paracent* or drain* )

AND

(MH "Albumins+") OR ( (MH "Plasma Substitutes+") OR (MH "Colloids+") ) OR (MH "Diuretics+") OR (MH "Dextrans") OR TI ( albumin* or diuretic* or colloid* or dextran* or polygeline* or reinfusion* or plasma substitute* or ascites fluid* or haemaccel or haemacel or hemaccel or hydroxy starch* or volume expander* or plasma expander* ) OR AB ( albumin* or diuretic* or colloid* or dextran* or polygeline* or reinfusion* or plasma substitute* or ascites fluid* or haemaccel or haemacel or hemaccel or hydroxy starch* or volume expander* or plasma expander* )

WHO ICTRP: 0 new record

dose albumin liver cirrhosis

clinicaltrial.gov: 0 records

dose albumin liver cirrhosis

### Supplementary table 3. Risk of bias

**Cochrane Risk of Bias Tool for Randomized Controlled Trials**

| **RANDOM SEQUENCE GENERATION Selection bias (biased allocation to interventions) due to inadequate generation of a randomised sequence.** | |
| --- | --- |
| **Criteria for a judgment of ‘Low risk’ of bias.** | **The investigators describe a random component in the sequence generation process such as:**   - **Referring to a random number table;** - **Using a computer random number generator;** - **Coin tossing;** - **Shuffling cards or envelopes;** - **Throwing dice;** - **Drawing of lots;** - **Minimization*. *Minimization may be implemented without a random element, and this is considered to be equivalent to being random.** |
| **Criteria for the judgment of ‘High risk’ of bias.** | **The investigators describe a non-random component in the sequence generation process. Usually, the description would involve some systematic, non-random approach, for example:**   - **Sequence generated by odd or even date of birth;** - **Sequence generated by some rule based on date (or day) of admission;** - **Sequence generated by some rule based on hospital or clinic record number. Other non-random approaches happen much less frequently than the systematic approaches mentioned above and tend to be obvious. They usually involve judgement or some method of non-random categorization of participants, for example:** - **Allocation by judgement of the clinician;** - **Allocation by preference of the participant;** - **Allocation based on the results of a laboratory test or a series of tests;** - **Allocation by availability of the intervention.** |
| **Criteria for the judgment of ‘Unclear risk’ of bias.** | **Insufficient information about the sequence generation process to permit judgement of ‘Low risk’ or ‘High risk’.** |

| **ALLOCATION CONCEALMENT Selection bias (biased allocation to interventions) due to inadequate concealment of allocations prior to assignment.** | |
| --- | --- |
| **Criteria for a judgment of ‘Low risk’ of bias.** | **Participants and investigators enrolling participants could not foresee assignment because one of the following, or an equivalent method, was used to conceal allocation:**   - **Central allocation (including telephone, web-based and pharmacy- controlled randomization);** - **Sequentially numbered drug containers of identical appearance;** - **Sequentially numbered, opaque, sealed envelopes.** |
| **Criteria for the judgment of ‘High risk’ of bias.** | **Participants or investigators enrolling participants could possibly foresee assignments and thus introduce selection bias, such as allocation based on:**   - **Using an open random allocation schedule (e.g. a list of random numbers);** - **Assignment envelopes were used without appropriate safeguards (e.g. if envelopes were unsealed or non-opaque or not sequentially numbered);** - **Alternation or rotation;** - **Date of birth;** - **Case record number;** - **Any other explicitly unconcealed procedure.** |
| **Criteria for the judgment of ‘Unclear risk’ of bias.** | **Insufficient information to permit judgement of ‘Low risk’ or ‘High risk’. This is usually the case if the method of concealment is not described or not described in sufficient detail to allow a definite judgement – for example if the use of assignment envelopes is described, but it remains unclear whether envelopes were sequentially numbered, opaque and sealed.** |

**SELECTIVE REPORTING
Reporting bias due to selective outcome reporting.**

| **Criteria for a judgment of ‘Low risk’ of bias.** | **Any of the following:**   - **The study protocol is available and all of the study’s pre-specified (primary and secondary) outcomes that are of interest in the review have been reported in the pre-specified way;** - **The study protocol is not available but it is clear that the published reports include all expected outcomes, including those that were pre- specified (convincing text of this nature may be uncommon).** |
| --- | --- |
| **Criteria for the judgment of ‘High risk’ of bias.** | **Any one of the following:**   - **Not all of the study’s pre-specified primary outcomes have been reported;** - **One or more primary outcomes is reported using measurements, analysis methods or subsets of the data (e.g. subscales) that were not pre-specified;** - **One or more reported primary outcomes were not pre-specified** |

|  | **(unless clear justification for their reporting is provided, such as an**  **unexpected adverse effect);**   - **One or more outcomes of interest in the review are reported incompletely so that they cannot be entered in a meta-analysis;** - **The study report fails to include results for a key outcome that would be expected to have been reported for such a study.** |
| --- | --- |
| **Criteria for the judgment of ‘Unclear risk’ of bias.** | **Insufficient information to permit judgement of ‘Low risk’ or ‘High risk’. It is likely that the majority of studies will fall into this category.** |

**OTHER BIAS
Bias due to problems not covered elsewhere in the table.

Criteria for a judgment of ‘Low risk’ of bias. The study appears to be free of other sources of bias.**

| **Criteria for the judgment of ‘High risk’ of bias.** | **There is at least one important risk of bias. For example, the study:**   - **Had a potential source of bias related to the specific study design used; or** - **Has been claimed to have been fraudulent; or** - **Had some other problem.** | |
| --- | --- | --- |
| **Criteria for the judgment of ‘Unclear risk’ of bias.** | **There may be a risk of bias, but there is either:**   - **Insufficient information to assess whether an important risk of bias exists; or** - **Insufficient rationale or evidence that an identified problem will introduce bias.** | |
| **BLINDING OF PARTICIPANTS AND PERSONNEL Performance bias due to knowledge of the allocated interventions by participants and personnel during the study.** | | |
| **Criteria for a judgment of ‘Low risk’ of bias.** | | **Any one of the following:**   - **No blinding or incomplete blinding, but the review authors judge that the outcome is not likely to be influenced by lack of blinding;** - **Blinding of participants and key study personnel ensured, and unlikely that the blinding could have been broken.** |
| **Criteria for the judgment of ‘High risk’ of bias.** | | **Any one of the following:**   - **No blinding or incomplete blinding, and the outcome is likely to be influenced by lack of blinding;** - **Blinding of key study participants and personnel attempted, but likely that the blinding could have been broken, and the outcome is likely to be influenced by lack of blinding.** |
| **Criteria for the judgment of ‘Unclear risk’ of bias.** | | **Any one of the following:**   - **Insufficient information to permit judgment of ‘Low risk’ or ‘High risk’;** - **The study did not address this outcome.** |

**BLINDING OF OUTCOME ASSESSMENT
Detection bias due to knowledge of the allocated interventions by outcome assessors.**

| **Criteria for a judgment of ‘Low risk’ of bias.** | **Any one of the following:**   - **No blinding of outcome assessment, but the review authors judge that the outcome measurement is not likely to be influenced by lack of blinding;** - **Blinding of outcome assessment ensured, and unlikely that the blinding could have been broken.** |
| --- | --- |
| **Criteria for the judgment of ‘High risk’ of bias.** | **Any one of the following:**   - **No blinding of outcome assessment, and the outcome measurement is likely to be influenced by lack of blinding;** - **Blinding of outcome assessment, but likely that the blinding could have been broken, and the outcome measurement is likely to be influenced by lack of blinding.** |
| **Criteria for the judgment of ‘Unclear risk’ of bias.** | **Any one of the following:**   - **Insufficient information to permit judgment of ‘Low risk’ or ‘High risk’;** - **The study did not address this outcome.** |

**INCOMPLETE OUTCOME DATA
Attrition bias due to amount, nature or handling of incomplete outcome data.**

| **Criteria for a judgment of ‘Low risk’ of bias.** | **Any one of the following:**   - **No missing outcome data;** - **Reasons for missing outcome data unlikely to be related to true outcome (for survival data, censoring unlikely to be introducing bias);** - **Missing outcome data balanced in numbers across intervention groups, with similar reasons for missing data across groups;** - **For dichotomous outcome data, the proportion of missing outcomes compared with observed event risk not enough to have a clinically relevant impact on the intervention effect estimate;** - **For continuous outcome data, plausible effect size (difference in means or standardized difference in means) among missing outcomes not enough to have a clinically relevant impact on observed effect size;** - **Missing data have been imputed using appropriate methods.** |
| --- | --- |
| **Criteria for the judgment of ‘High risk’ of bias.** | **Any one of the following:**   - **Reason for missing outcome data likely to be related to true outcome, with either imbalance in numbers or reasons for missing data across intervention groups;** - **For dichotomous outcome data, the proportion of missing outcomes compared with observed event risk enough to induce clinically relevant bias in intervention effect estimate;** |

|  | - **For continuous outcome data, plausible effect size (difference in means or standardized difference in means) among missing outcomes enough to induce clinically relevant bias in observed effect size;** - **‘As-treated’ analysis done with substantial departure of the intervention received from that assigned at randomization;** - **Potentially inappropriate application of simple imputation.** |
| --- | --- |
| **Criteria for the judgment of ‘Unclear risk’ of bias.** | **Any one of the following:**   - **Insufficient reporting of attrition/exclusions to permit judgement of ‘Low risk’ or ‘High risk’ (e.g. number randomized not stated, no reasons for missing data provided);** - **The study did not address this outcome.** |

**Thresholds for Converting the Cochrane Risk of Bias Tool to AHRQ Standards (Good, Fair, and Poor)**

**Good quality: All criteria met (i.e. low for each domain)**

**Using the Cochrane ROB tool, it is possible for a criterion to be met even when the element was technically not part of the method. For instance, a judgment that knowledge of the allocated interventions was adequately prevented can be made even if the study was not blinded, if EPC team members judge that the outcome and the outcome measurement are not likely to be influenced by lack of blinding.**

**Fair quality: One criterion not met (i.e. high risk of bias for one domain) or two criteria unclear, and the assessment that this was unlikely to have biased the outcome, and there is no known important limitation that could invalidate the results**

**Poor quality: One criterion not met (i.e. high risk of bias for one domain) or two criteria unclear, and the assessment that this was likely to have biased the outcome, and there are important limitations that could invalidate the results**

**Poor quality: Two or more criteria listed as high or unclear risk of bias**

### Supplementary table 4. Cochrane checklist RCT’s

| Validiteit |  |  |  |
| --- | --- | --- | --- |
| Item | + | - | ? |
| Was de toewijzing van de interventie aan de patienten gerandomiseerd? |  |  |  |
| Was degene die patienten insluit niet op de hoogte van de randomisatievolgorde? |  |  |  |
| Waren de patienten en de behandelaars geblindeerd voor de behandeling? |  |  |  |
| Waren de effectbeoordelaars geblindeerd voor de behandeling? |  |  |  |
| Waren de groepen aan het begin van de trial vergelijkbaar? |  |  |  |
| Indien nee: is hiervoor in de analyses gecorrigeerd? |  |  |  |
| Is van een voldoende proportie van alle ingesloten patiënten een volledige follow-up beschikbaar? |  |  |  |
| Indien nee: is selectieve loss-to-follow-up voldoende uitgesloten? |  |  |  |
| Zijn alle ingesloten patiënten geanalyseerd in de groep waarin ze waren gerandomiseerd? |  |  |  |
| Zijn de groepen, afgezien van de interventie, gelijk behandeld? |  |  |  |
| Is selectieve publicatie van uitkomsten voldoende uitgesloten? |  |  |  |
| Is ongewenste invloed van sponsoren/academic bias voldoende uitgesloten? |  |  |  |

### Supplementary table 5. Quality assessment

Newcastle-Ottawa Quality Assessment Form for Cohort Studies

Note: A study can be given a maximum of one star for each numbered item within the Selection and Outcome categories. A maximum of two stars can be given for Comparability.

Selection

- 1) Representativeness of the exposed cohort
  - a) Truly representative (one star)
  - b) Somewhat representative (one star)
  - c) Selected group
  - d) No description of the derivation of the cohort
- 2) Selection of the non-exposed cohort
  - a) Drawn from the same community as the exposed cohort (one star)
  - b) Drawn from a different source
  - c) No description of the derivation of the non exposed cohort
- 3) Ascertainment of exposure
  - a) Secure record (e.g., surgical record) (one star)
  - b) Structured interview (one star)
  - c) Written self report
  - d) No description
  - e) Other
- 4) Demonstration that outcome of interest was not present at start of study

a) Yes (one star) b) No

Comparability

1) Comparability of cohorts on the basis of the design or analysis controlled for confounders

- a) The study controls for age, sex and marital status (one star)
- b) Study controls for other factors (list) _________________________________ (one star)
- c) Cohorts are not comparable on the basis of the design or analysis controlled for confounders

Outcome

- 1) Assessment of outcome
  - a) Independent blind assessment (one star)
  - b) Record linkage (one star)
  - c) Self report
  - d) No description
  - e) Other
- 2) Was follow-up long enough for outcomes to occur

a) Yes (one star)

b) No
Indicate the median duration of follow-up and a brief rationale for the assessment above:____________________

3) Adequacy of follow-up of cohorts

- a) Complete follow up- all subject accounted for (one star)
- b) Subjects lost to follow up unlikely to introduce bias- number lost less than or equal to 20% or description of those lost
  suggested no different from those followed. (one star)
- c) Follow up rate less than 80% and no description of those lost
- d) No statement

Thresholds for converting the Newcastle-Ottawa scales to AHRQ standards (good, fair, and poor):

Good quality: 3 or 4 stars in selection domain AND 1 or 2 stars in comparability domain AND 2 or 3 stars in outcome/exposure domain

Fair quality: 2 stars in selection domain AND 1 or 2 stars in comparability domain AND 2 or 3 stars in outcome/exposure domain

Poor quality: 0 or 1 star in selection domain OR 0 stars in comparability domain OR 0 or 1 stars in outcome/exposure domain

### Supplementary table 6. PRISMA 2020 checklist

| **Section and Topic** | **Item #** | **Checklist item** | **Location where item is reported** |
| --- | --- | --- | --- |
| **TITLE** | | |  |
| Title | 1 | Identify the report as a systematic review. | Title |
| **ABSTRACT** | | |  |
| Abstract | 2 | See the PRISMA 2020 for Abstracts checklist. | Material and methods |
| **INTRODUCTION** | | |  |
| Rationale | 3 | Describe the rationale for the review in the context of existing knowledge. | Introduction |
| Objectives | 4 | Provide an explicit statement of the objective(s) or question(s) the review addresses. | Introduction |
| **METHODS** | | |  |
| Eligibility criteria | 5 | Specify the inclusion and exclusion criteria for the review and how studies were grouped for the syntheses. | Search strategy and study selection |
| Information sources | 6 | Specify all databases, registers, websites, organisations, reference lists and other sources searched or consulted to identify studies. Specify the date when each source was last searched or consulted. | Search strategy and study selection |
| Search strategy | 7 | Present the full search strategies for all databases, registers and websites, including any filters and limits used. | Search strategy and study selection & supplements |
| Selection process | 8 | Specify the methods used to decide whether a study met the inclusion criteria of the review, including how many reviewers screened each record and each report retrieved, whether they worked independently, and if applicable, details of automation tools used in the process. | Search strategy and study selection |
| Data collection process | 9 | Specify the methods used to collect data from reports, including how many reviewers collected data from each report, whether they worked independently, any processes for obtaining or confirming data from study investigators, and if applicable, details of automation tools used in the process. | Search strategy and study selection |
| Data items | 10a | List and define all outcomes for which data were sought. Specify whether all results that were compatible with each outcome domain in each study were sought (e.g. for all measures, time points, analyses), and if not, the methods used to decide which results to collect. | Data extraction |
|  | 10b | List and define all other variables for which data were sought (e.g. participant and intervention characteristics, funding sources). Describe any assumptions made about any missing or unclear information. | Data extraction |
| Study risk of bias assessment | 11 | Specify the methods used to assess risk of bias in the included studies, including details of the tool(s) used, how many reviewers assessed each study and whether they worked independently, and if applicable, details of automation tools used in the process. | Quality Assessment |
| Effect measures | 12 | Specify for each outcome the effect measure(s) (e.g. risk ratio, mean difference) used in the synthesis or presentation of results. | Data extraction |
| Synthesis methods | 13a | Describe the processes used to decide which studies were eligible for each synthesis (e.g. tabulating the study intervention characteristics and comparing against the planned groups for each synthesis (item #5)). | Data extraction |
|  | 13b | Describe any methods required to prepare the data for presentation or synthesis, such as handling of missing summary statistics, or data conversions. | NA |
|  | 13c | Describe any methods used to tabulate or visually display results of individual studies and syntheses. | NA |
|  | 13d | Describe any methods used to synthesize results and provide a rationale for the choice(s). If meta-analysis was performed, describe the model(s), method(s) to identify the presence and extent of statistical heterogeneity, and software package(s) used. | NA |
|  | 13e | Describe any methods used to explore possible causes of heterogeneity among study results (e.g. subgroup analysis, meta-regression). | NA |
|  | 13f | Describe any sensitivity analyses conducted to assess robustness of the synthesized results. | NA |
| Reporting bias assessment | 14 | Describe any methods used to assess risk of bias due to missing results in a synthesis (arising from reporting biases). | Quality Assessment |
| Certainty assessment | 15 | Describe any methods used to assess certainty (or confidence) in the body of evidence for an outcome. | Quality assessment |
| **RESULTS** | | |  |
| Study selection | 16a | Describe the results of the search and selection process, from the number of records identified in the search to the number of studies included in the review, ideally using a flow diagram. | Study selection  Ongoing trials |
|  | 16b | Cite studies that might appear to meet the inclusion criteria, but which were excluded, and explain why they were excluded. | Study selection  Ongoing trials |
| Study characteristics | 17 | Cite each included study and present its characteristics. | Results |
| Risk of bias in studies | 18 | Present assessments of risk of bias for each included study. | Quality assessment |
| Results of individual studies | 19 | For all outcomes, present, for each study: (a) summary statistics for each group (where appropriate) and (b) an effect estimate and its precision (e.g. confidence/credible interval), ideally using structured tables or plots. | Study results |
| Results of syntheses | 20a | For each synthesis, briefly summarise the characteristics and risk of bias among contributing studies. | Quality assessment |
|  | 20b | Present results of all statistical syntheses conducted. If meta-analysis was done, present for each the summary estimate and its precision (e.g. confidence/credible interval) and measures of statistical heterogeneity. If comparing groups, describe the direction of the effect. | NA |
|  | 20c | Present results of all investigations of possible causes of heterogeneity among study results. | NA |
|  | 20d | Present results of all sensitivity analyses conducted to assess the robustness of the synthesized results. | NA |
| Reporting biases | 21 | Present assessments of risk of bias due to missing results (arising from reporting biases) for each synthesis assessed. | Quality assessment |
| Certainty of evidence | 22 | Present assessments of certainty (or confidence) in the body of evidence for each outcome assessed. | Study results |
| **DISCUSSION** | | |  |
| Discussion | 23a | Provide a general interpretation of the results in the context of other evidence. | Discussion |
|  | 23b | Discuss any limitations of the evidence included in the review. | Discussion |
|  | 23c | Discuss any limitations of the review processes used. | Strengths and limitations |
|  | 23d | Discuss implications of the results for practice, policy, and future research. | Future studies |
| **OTHER INFORMATION** | | |  |
| Registration and protocol | 24a | Provide registration information for the review, including register name and registration number, or state that the review was not registered. | Study design |
|  | 24b | Indicate where the review protocol can be accessed, or state that a protocol was not prepared. | Study design |
|  | 24c | Describe and explain any amendments to information provided at registration or in the protocol. | Study design |
| Support | 25 | Describe sources of financial or non-financial support for the review, and the role of the funders or sponsors in the review. | Financial support statement |
| Competing interests | 26 | Declare any competing interests of review authors. | Conflict of interest statement |
| Availability of data, code and other materials | 27 | Report which of the following are publicly available and where they can be found: template data collection forms; data extracted from included studies; data used for all analyses; analytic code; any other materials used in the review. | Supplementary |

*From:*  Page MJ, McKenzie JE, Bossuyt PM, Boutron I, Hoffmann TC, Mulrow CD, et al. The PRISMA 2020 statement: an updated guideline for reporting systematic reviews. BMJ 2021;372:n71. doi: 10.1136/bmj.n71. This work is licensed under CC BY 4.0. To view a copy of this license, visit <https://creativecommons.org/licenses/by/4.0/>

### Supplementary reference

1. Sterne JAC, Savović J, Page MJ, Elbers RG, Blencowe NS, Boutron I, et al. RoB 2: a revised tool for assessing risk of bias in randomised trials. Bmj. 2019;366:l4898.
2. Offringa M, Assendelft, W. J. J., Scholten, R. J. P. M., Leeflang, M. M. G., Bossuyt, P. M. M., Groenwold, R. H. H., Reitsma, J. B., van de Laar, F. A., van Puijenbroek, E. P., Broeders, M. J. M., van der Graaf, Y., Terwee, C. B., de Vet, H. C. W., Lucassen, P. L. B. J., Reis, R., & Dees, M. . Kritisch beoordelen van een artikel over primair onderzoek. In Inleiding in evidence-based medicine. Bohn Stafleu van Loghum.; 2018. p. 29–119.
3. Ottawa hospital research institute.: Ohri.Ca.; 2024.
4. Page MJ, McKenzie JE, Bossuyt PM, Boutron I, Hoffmann TC, Mulrow CD, et al. The PRISMA 2020 statement: an updated guideline for reporting systematic reviews. Bmj. 2021;372:n71.
